# Biologically Active Polymers from Spontaneous Carotenoid Oxidation: A New Frontier in Carotenoid Activity

**DOI:** 10.1371/journal.pone.0111346

**Published:** 2014-10-31

**Authors:** James B. Johnston, James G. Nickerson, Janusz Daroszewski, Trevor J. Mogg, Graham W. Burton

**Affiliations:** 1 National Research Council of Canada, Charlottetown, Prince Edward Island, Canada; 2 Avivagen Inc., Charlottetown, Prince Edward Island, Canada; 3 Avivagen Inc., Ottawa, Ontario, Canada; Hokkaido University, Japan

## Abstract

In animals carotenoids show biological activity unrelated to vitamin A that has been considered to arise directly from the behavior of the parent compound, particularly as an antioxidant. However, the very property that confers antioxidant activity on some carotenoids in plants also confers susceptibility to oxidative transformation. As an alternative, it has been suggested that carotenoid oxidative breakdown or metabolic products could be the actual agents of activity in animals. However, an important and neglected aspect of the behavior of the highly unsaturated carotenoids is their potential to undergo addition of oxygen to form copolymers. Recently we reported that spontaneous oxidation of ß-carotene transforms it into a product dominated by ß-carotene-oxygen copolymers. We now report that the polymeric product is biologically active. Results suggest an overall ability to prime innate immune function to more rapidly respond to subsequent microbial challenges. An underlying structural resemblance to sporopollenin, found in the outer shell of spores and pollen, may allow the polymer to modulate innate immune responses through interactions with the pattern recognition receptor system. Oxygen copolymer formation appears common to all carotenoids, is anticipated to be widespread, and the products may contribute to the health benefits of carotenoid-rich fruits and vegetables.

## Introduction

Fruit and vegetable intake-based epidemiological studies relating to the incidence of chronic diseases such as cancer and heart disease [1] have created considerable interest in potential non-vitamin A benefits of carotenoids and of ß-carotene in particular. Various possible mechanisms operating at the functional, cellular and molecular levels have been proposed [1], [2], [3], [4]. Of these, a possible antioxidant function [1], [5] initially attracted much interest [4]. However, the lack of support or even failure of early human intervention trials [6], [7], [8] cast serious doubt upon the value of pharmaceutical-level ß-carotene supplementation for ameliorating chronic diseases. Furthermore, the recent observation that antioxidant supplementation enhances cancer progression in mice [9] undermines an antioxidant role.

Actual demonstrated non-vitamin A health benefits of carotenoids in animals point to involvement of immune function [2], [10], [11], [12], [13], [14]. It has been suggested carotenoids can participate in and modulate processes involving reactive oxygen species [2], [3], [12]. This behavior would still be consistent with the dual antioxidant/pro-oxidant character of carotenoids inherent in the extensive system of conjugated double bonds [4], [5]. In this scenario, carotenoid oxidative breakdown products [15] or their metabolites [16] have been suggested to be the actual bioactive agents. However, progress in this area has been hampered by a lack of identified candidate compounds.

Recently, we reported the discovery that spontaneous oxidation of ß-carotene is dominated not by cleavage reactions but by *addition* of oxygen to form potentially bioactive, oxygen-rich, ß-carotene-oxygen copolymers [17]. We also found that this dominance appears to be common to most, if not all, carotenoid compounds, even early on in the oxidation. Given this finding and the ubiquity of carotenoids and their susceptibility to oxidation during exposure to air, it is anticipated that carotenoid-oxygen co-polymers would occur naturally in a variety of situations. In this regard and as an example, we have noted [17] strong similarities in the elemental compositions and infrared spectra of the products from fully oxidized ß-carotene and lycopene on one hand, and, on the other, sporopollenin, an almost chemically and biochemically intractable polymeric component of the highly robust outer walls (exines) of pollens and spores [18]. We surmised that the carotenoid-oxygen copolymer compounds are similar to an early stage or precursor form of the highly elaborated, naturally occurring sporopollenin exine, sharing a common underlying chemical motif. Indeed Brooks and Shaw first proposed carotenoid-oxygen emulsion copolymerization is a key, early step in the formation of sporopollenin [18], [19], [20], [21].

Many animal species would be expected to be exposed to these compounds in low and varying amounts in foods and the environment. The question arises: is this previously overlooked class of carotenoid-derived oxygen copolymers biologically active?

As a first step in addressing this question we took fully autoxidized ß-carotene (OxC-beta) as a representative cross-section of carotenoid oxidation products [17] to test for evidence of biological activity *in vitro*. OxC-beta, containing more than 30% oxygen by weight, has a defined product composition comprising ß-carotene oxygen copolymers (85% w/w) and minor amounts of short chain, mostly familiar, norisoprenoid cleavage compounds. Vitamin A and higher molecular weight norisoprenoid compounds are absent. Because ß-carotene also is absent, any biological activity must arise directly from non-vitamin A oxidation products and necessarily precludes any involvement of ß-carotene as an antioxidant. In a preliminary evaluation of biological activity using QRT-PCR, we reported OxC-beta increases expression of several genes associated with pathogen recognition and host defense, including toll-like receptors subtype 2 and 4 (TLR-2, TLR-4), CD14, and several genes involved in the TLR signaling pathway [17].

An earlier, preliminary feeding trial with broiler chickens to evaluate the potential of OxC-beta as a non-antibiotic enhancer of food animal productivity gave results that are consistent with an immune priming effect. We therefore carried out studies, reported here, that undertook to (a) directly establish OxC-beta’s effect upon measures of innate immune function associated with host detection and response to bacterial pathogens and (b) identify the polymer fraction as the main source of activity within OxC-beta and other oxidized carotenoids.

## Results, Discussion and Conclusions

**Figure 1:**
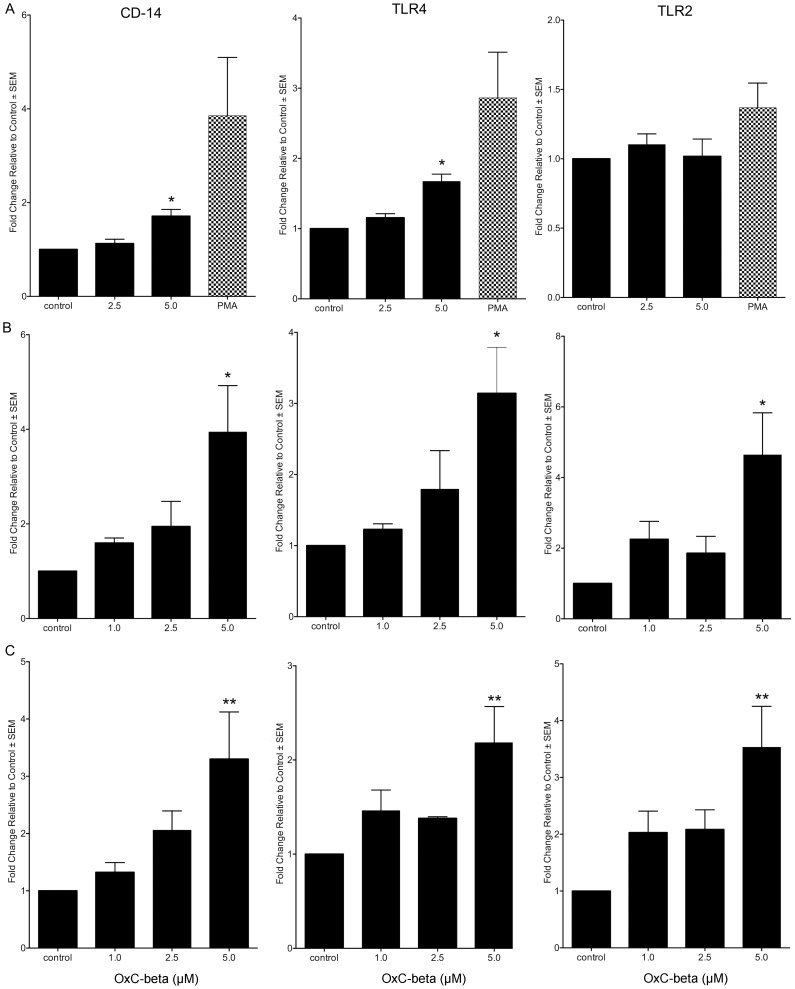
Effect of OxC-beta on CD14, TLR-4, and TLR-2 levels *in vitro*. Human THP-1 monocytes (A), fibroblasts (B), and endothelial cells (C), were treated with the indicated concentrations of OxC-beta or vehicle control (DMSO) for 24 hours. Immune receptor content was measured 24 hours post-treatment by FACS analysis. OxC-beta-induced increase in receptor level was assessed relative to untreated control cells using a one-way analysis of variance with Tukey’s post test for multiple comparisons. DMSO had no effect on receptor level (result not shown). Phorbol myristate acetate (PMA) was used as a positive control in experiments with THP-1 cells (hatched bars).

**Figure 2:**
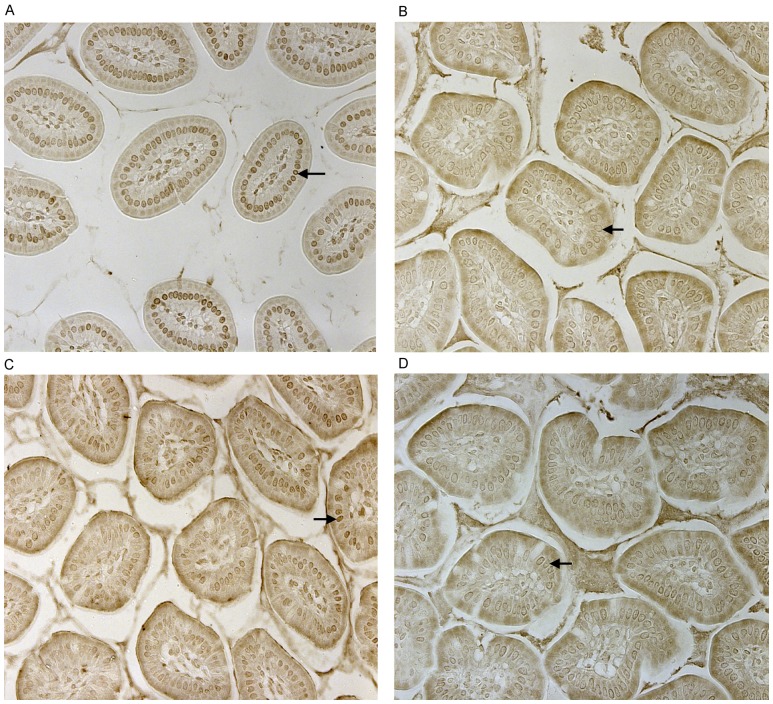
CD14 and TLR-4 staining in gut epithelial cells. Balb/c mice were not supplemented (control) or supplemented daily by oral gavage with OxC-beta (10 mg/kg). After 4 weeks, intestinal tissues were harvested and CD14 and TLR-4 expression was determined by immunocytochemistry. Increased CD14 (A) and TLR-4 (C) expression is readily apparent in epithelial cells in the OxC-beta-supplemented animals compared to the controls receiving vehicle alone (B and D, respectively). Arrows indicate the location of enterocytes within the cross section of microvilli. Magnification 40x.

Flow cytometry confirms the earlier PCR assay results [17], showing up-regulation of TLR and CD14 expression. Treatment with OxC-beta increases plasma membrane content of TLRs and CD14 in several cultured cell types, including monocytes, fibroblasts and endothelial cells (Fig. 1). In monocytes OxC-beta (5.0 µM) significantly increases plasma membrane content of both CD14 and TLR-4 (1.7-fold for both), without altering TLR-2 levels (Fig. 1A). The effect on fibroblasts is more pronounced (Fig. 1B). OxC-beta (5.0 µM) induces increases in CD14 and TLR-4 of 3.9 and 3.1-fold, respectively. TLR-2 also increases significantly by 4.6-fold. CD14, TLR-4 and TLR-2 levels also increase significantly in endothelial cells (Fig. 2C): treatment with 5.0 µM OxC-beta produces increases of 3.3, 2.2 and 3.5-fold, respectively. These results indicate that the previously reported PCR gene expression results are reflected at the protein level.

The *in vitro* receptor results are corroborated *in vivo.* Mice fed OxC-beta show increased intestinal immune receptor content. Oral supplementation of OxC-beta at 10 mg/kg body weight daily for 2 or 4 weeks show marked increases in levels of CD14 and TLR-4 in the small intestine (see Fig. 2 for results at 4 weeks). Furthermore, OxC-beta’s effect on receptors appears dose dependent: the increase in receptors for 1 mg/kg, though apparent, was less pronounced as qualitatively indicated by the intensity of staining (results not shown).

The ability to modulate host-innate-responsiveness to bacterial infection has been assessed by evaluating OxC-beta’s effect upon the early stage cytokine response and phagocytic activity of monocytes under both naïve and simulated challenge situations. Given OxC-beta’s ability to increase TLR-4 and CD14 levels and the critical role that these moieties play in the receptor complex that detects bacterial lipopolysaccharide (LPS), we used an LPS challenge to mimic bacterial infection *in vitro*.

OxC-beta treatment of naïve monocytes has no apparent effect on the level of various cytokines, including TNFα, IL-1β, IL-6, IFNγ, MCP-1 (CCL-2), and IL-8 (results not shown). However, when OxC-beta treatment is followed by an LPS-challenge the pretreatment potentiates the LPS-induced increase in the production of several cytokines, including TNFα (25%), IL-6 (48%), and IL-1β (40%), relative to LPS- challenge alone (Table 1). TNFα and IL-1β are pivotal pleiotropic cytokines directing multiple facets of host response to infection whereas IL-6 is an important mediator of the acute phase response and is required for maintaining microbial resistance.

**Table 1:** Cytokine expression following OxC-beta treatment and LPS challenge.

Conc.^2^	TNFα		IL-6		IL-1β		IFNγ	
µM	pg/mL^3^	Fold^4^	pg/mL^3^	Fold^4^	pg/mL^3^	Fold^4^	pg/mL^3^	Fold^4^
0.0	1471±171	1.00	37.7±1.9	1.00	1194±46	1.00	20.9±0.6	1.00
0.1	1840±3**^#^**	1.25	49.4±3.9^*^	1.31	1425±117	1.19	21.0±0.1	1.00
0.5	1846±7**^#^**	1.25	56.1±2.8^*^	1.48	1669±78^*^	1.40	20.5±2.7	0.98
1.0	1832±0.4**^#^**	1.24	37.8±2.2	1.00	1065±95	0.89	19.4±3.7	0.93

All cells were challenged with 15 ng/mL LPS.

2 OxC-beta concentration expressed using the molecular weight of ß-carotene.

3 Values determined by reference standards included in each assay.

4 Fold change relative to untreated cells (no OxC-beta).

# *p*<0.005, * *p*<0.05 (Student’s *t*-test versus untreated).

**Figure 3:**
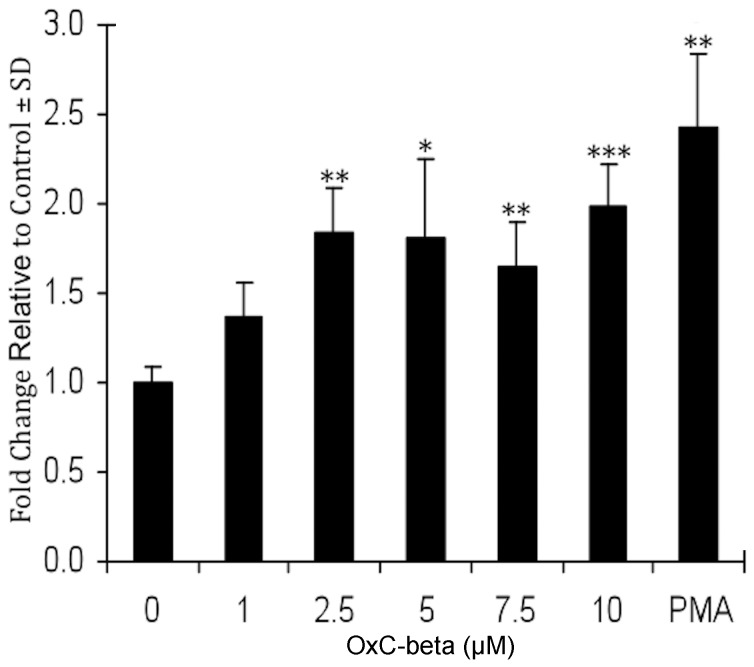
Phagocytosis in OxC-beta treated and LPS-stimulated THP-1 cells. THP-1 monocytes were incubated with the indicated concentration of OxC-beta or DMSO control for 24 hours before being treated with LPS (15 ng/mL). Phagocytosis was evaluated 24 hours after LPS stimulation. Values represent fold changes relative to controls. PMA was used at 25 ng/mL. * p<0.05, ** p<0.02, *** p<0.002, Student’s t-test versus controls.

At the level of monocyte function, OxC-beta treatment increases the phagocytic activity of monocytes. The effect is most prominent under LPS-challenge conditions where pretreatment with OxC-beta increases activity to a level comparable to the positive control, phorbol myristate acetate (PMA, Fig. 3). By contrast the effect of OxC-beta on phagocytic activity in naïve monocytes is more modest (result not shown).

Taken together the results on immune receptor levels, cytokine levels, and phagocytic activity provide mechanistic and function-based evidence that OxC-beta modulates innate immunity in a biologically relevant way. Furthermore, the results suggest that the overall impact would be to prime the innate immune system to more rapidly respond to subsequent challenges. The fact that OxC-beta has little to no apparent effect on cytokine levels and phagocytic activity in the absence of a challenge distinguishes it from traditional immune stimulants, which directly trigger an inflammatory immune response.

**Figure 4:**
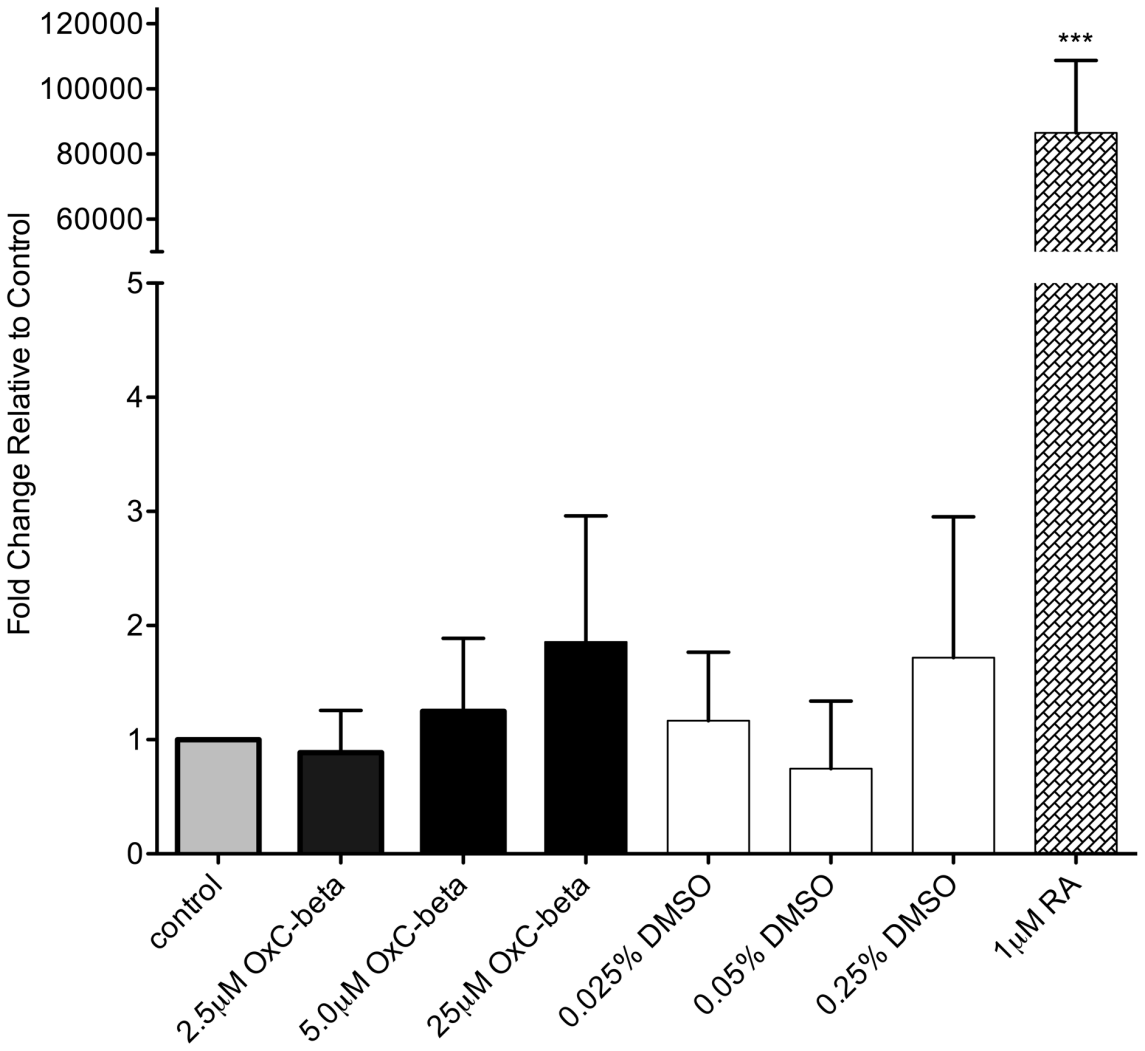
Effect of OxC-beta treatment on CYP26A gene expression in MCF-7 cells. Cells were incubated in the presence of the indicated concentrations of OxC-beta or vehicle control for 24 hours. For vehicle controls the concentrations of DMSO used were equivalent to the concentration of DMSO in the OxC-beta treatment groups, i.e., control groups labeled as 0.025%, 0.05% and 0.25% DMSO had the same DMSO concentration (v/v) as was used in the 2.5 µM, 5.0 µM and 25 µM OxC-beta treatment groups, respectively. Untreated cells were used as negative controls and cells treated with 1.0 µM of *all trans* retinoic acid (RA) served as positive controls. CYP26A gene expression was measured relative to β-actin using quantitative real-time PCR with the two standard curve method. Bars represent the mean ratio of CYP26A expression relative to β-actin from 4 separate experiments. Error bars represent the standard error of the mean. One-way ANOVA with Tukey’s test for multiple comparisons indicated no significant difference in relative CYP26A expression between cells treated with OxC-beta, DMSO, or untreated control cells. Treatment with 1.0 µM RA induced a significant increase in CYP26A gene expression compared to all other treatment groups. CYP26A expression ratios are shown calibrated to untreated control cells. *** p<0.001.

Given that activation of the retinoic acid receptor (RAR) pathway is known to affect immune function [22], it was important to rule out the possibility that unidentified compounds within OxC-beta possessed RAR agonist activity. Treatment of MCF-7 cells with OxC-beta failed to induce expression of CYP26A, a known RAR responsive gene [23] (Fig. 4). This result is consistent with the reported absence of retinoic acid and closely related compounds in OxC-beta [17].

**Figure 5:**
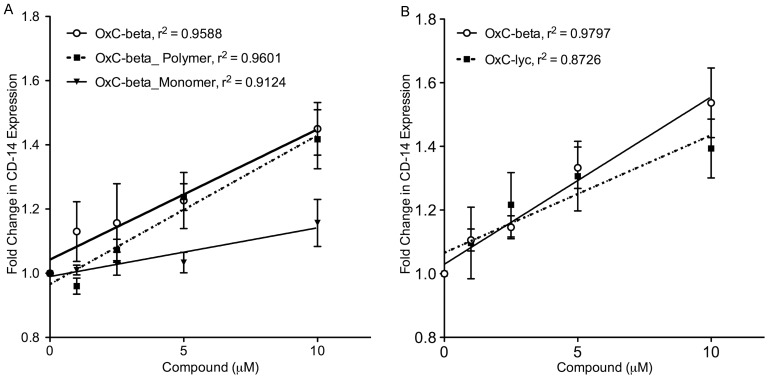
Determination of activities relative to OxC-beta of (A) OxC-beta polymer and monomer fractions, and (B) oxidized lycopene (OxC-lyc), using a CD14 receptor expression assay. THP-1 cells were treated for 24 hours with the indicated concentrations of compounds. CD14 expression was quantified using FACS analysis. The effect of each compound is shown relative to untreated cells. Points represent the mean and standard error from three separate experiments. (A) Correlation analysis indicates a significant dose effect for each compound on CD14 expression with p-values of 0.0036 for OxC-beta, 0.0034 for the polymer, and 0.0113 for the monomer. Comparison of the relative activity of each compound indicates that the monomer is significantly less active than the polymer (p<0.001) and OxC-beta (p<0.01) while there is no significant difference between the activities of the polymer and OxC-beta. The apparent activity of the monomer may be due to the presence of residual polymers that could not be completely removed from the monomer fraction. (B) OxC-lyc also had a significant dose effect on CD14 surface content (p = 0.020) that was not significantly different from the effect of OxC-beta.

Next we turned our attention towards identifying the compound(s) present in OxC-beta responsible for the observed immunological activity. A FACS assay based on OxC-beta’s ability to up-regulate CD14 in monocytes indicates the polymeric fraction is the source of activity. Treatment with equal amounts of OxC-beta or the isolated polymeric fraction induced significant and comparable increases in the surface content of monocyte CD14 relative to untreated control cells (Fig. 5A). The monomer fraction was much less active relative to either OxC-beta or the polymer, especially considering that OxC-beta and the polymer and monomer fractions were each compared over similar concentration ranges, with the monomer fraction concentration being approximately 7-fold higher relative to its actual level of 15% in OxC-beta. Any activity in the monomer fraction is attributed to the presence of lower molecular weight residual copolymer compounds that were not completely removed in the separation process. The CD14 FACS assay also shows fully oxidized lycopene (OxC-lyc) and OxC-beta have essentially the same up-regulating activity (Fig. 5B). Given OxC-lyc also is predominantly composed of oxygen co-polymers [17], it is reasonable to conclude these too are the source of activity.

The similarity of the activities of OxC-beta and OxC-lyc also makes it unlikely that the minor amounts of norisoprenoid compounds present in each, together with the dissimilarity of their composition, contribute any significant activity. The most abundant norisoprenoid in OxC-beta, geronic acid (2.4%) [17], is inactive (results not shown) and is not present in OxC-lyc. The next most abundant compounds in OxC-beta, ß-ionone-5,6-epoxide (1.2%), dihydroactinidiolide (1.1%) and ß-ionone (0.8%) [17], also are not found in OxC-lyc, as applies for any other norisoprenoid compounds derived from the cyclohexyl rings present in ß-carotene but absent in lycopene. Although several aldehydes have been identified in OxC-beta, they are present at very low levels, for example, 2-methyl-6-oxo-2,4-heptadienal (0.23%) [17]. The numerous other norisoprenoid compounds present in OxC-beta are present at much less than 1% levels, making it unlikely they make any significant contribution to activity, individually or collectively, especially in light of the data for the polymeric fraction presented in Fig. 5A.

The CD14 assay results have two-fold significance. Firstly, they demonstrate for the first time that carotenoid oxygen copolymers, the dominant products of spontaneous carotenoid oxidation, are biologically active. Given that OxC-beta, OxC-lyc and also fully oxidized canthaxanthin all have been found to be predominantly composed of oxygen copolymers [17], it appears reasonable to conclude that through oxidation the highly unsaturated backbone of carotenoids provides a route to a previously unrecognized class of biologically active, polymeric compounds. This very facile natural reaction opens up a new approach to understanding carotenoid non-vitamin A behavior, moving the focus from putative protective antioxidant *processes* to a discrete class of carotenoid oxidation *products* with unanticipated biological activities.

Secondly, the fact that the copolymers are capable of modulating innate immunity, a fundamental physiological system common to all animals, suggests the innate immune system has the ability to recognize structural elements within the polymer fraction and highlights the important role that these compounds potentially play in nature. Of note in this regard is the similar innate immune priming effect observed in an *in vitro* macrophage model [24] for carotenoid-containing spores of the common probiotic bacteria *Bacillus subtilus* [25], although it is not yet known if the activity arises from oxygen-carotenoid copolymers.

The absence of vitamin-A and higher homologs in OxC-beta and its inability to induce expression of an RAR responsive gene, together with the identification of the polymer as the active constituent in both OxC-beta and OxC-lyc, suggest that biological activity arises via a novel pathway. Given the apparent underlying structural similarity of the polymer to sporopollenin [17], [18], [19] and the likelihood of the latter to be associated with the reproductive structures of varied microbes (e.g., spores and pollen), it seems plausible that the innate immune system may have evolved mechanisms to recognize conserved molecular patterns contained in sporopollenin-like moieties.

The extent of the natural occurrence of oxidized carotenoids containing copolymeric components is not yet known, although sporopollenin, for example, in the form of spores and pollen is ubiquitous and widely distributed in the environment and foods [18], [26]. Even in small amounts the cumulative effects of frequent exposure to carotenoid copolymers may be sufficient to partly explain the epidemiological evidence for the benefits of diets rich in carotenoid-containing fruits and vegetables [1]. Also, the likely low level of oxidation products in ß-carotene supplements formulated to protect against oxidation may explain the lack of efficacy of high levels of supplementation in intervention trials [6], [7], [8]. The fact that intact sporopollenin exine shells have been shown to be readily absorbed across the gut wall and broken down quickly in blood [27] implies that carotenoid-oxygen copolymers would be systemically available for distribution to a wide range of tissues in the body. Indeed, the ability to convert spores and pollen into empty sporopollenin exine shells that can serve as vehicles for oral delivery of a variety of compounds into the blood is being developed as a novel technology for delivery of drugs and nutrients (http://www.sporomex.co.uk).

Indirect support for the systemic availability of OxC-beta is provided in a preliminary study to be reported elsewhere, with prima facie evidence of OxC-beta activity *in vivo* in exploratory animal feeding trials. Low part-per-million levels of OxC-beta in feed improved measures of production efficiency, i.e., feed conversion and growth, in both swine and broiler chickens. Interestingly, benefits in poultry were more pronounced in a *Clostridium perfringens* challenge model of necrotic enteritis, a finding consistent with the innate immune priming activity of OxC-beta reported above.

Further support for the systemic availability of orally-administered OxC-beta is provided in a study showing enhanced resolution of inflammation in a model of bovine respiratory disease [28]. This study illustrates *in vivo* the ability of OxC-beta to exert a moderating influence upon inflammation in the lung, an immunological activity that was suggested earlier by the *in vitro* results of a PCR gene expression array assay [17]. Both the innate immune priming effect and the anti-inflammatory/pro-resolution effects suggested by the PCR gene expression array results are supported by the results of multiple animal trials that are beyond the scope of this manuscript and will be the subject of future publications.

## Materials and Methods

### Preparation of OxC-beta and Isolation of Polymer and Monomer Fractions

The preparations of OxC-beta, its polymer and monomer fractions and OxC-lyc have been described [17].

### Cell Culture

THP-1, 1079SK-fibroblasts, and MCF-7 cell lines were obtained from American Type Cell Culture (ATCC). Human umbilical vein endothelial cells (HUVEC) were purchased from Life Technologies. All cell lines were cultured in complete media at 37°C with 5% CO_2_. For the human THP-1 monocyte line (ATCC TIB-202) complete media was RPMI-1640 media supplemented with 2 mM L-glutamine, 10 mM HEPES, 1.0 mM sodium pyruvate, 10% fetal bovine serum (FBS) and antibiotics. Fibroblasts (ATCC CRL-2097) were cultured in Eagle’s Minimum Essential Media (EMEM) supplemented with 0.1 mM non-essential amino acids, 0.1 mM sodium pyruvate, 10% FBS and antibiotics. MCF-7 cells (ATCC HTB-22) were cultured in EMEM supplemented with 10% FBS and antibiotics. HUVEC cells (Life Technologies C-003-5C) were cultured in Media 200 supplemented with low serum growth supplement (Life Technologies S00310) and antibiotics.

### Test Compound Preparation

10 mM (ß-carotene equivalents) stock solutions of OxC-beta, OxC-beta polymer fraction, OxC-beta monomer fraction, and OxC-lyc were prepared by dissolving 5.37 mg/mL of each compound in DMSO. Stock solutions were stored in 500 µL aliquots at −80°C. Working solutions (200 µM carotene equivalents) were prepared by further dilution of the stock with the appropriate basal media followed by filter sterilization (0.22 µm pore size). Cells were treated with the indicated concentration of each compound by further dilution of the 200 µM working stocks with the appropriate culture media.

### Measurement of OxC-beta Effect on Expression of CD14, TLR-4, and TLR-2 *In Vitro*

Cells were seeded in T-25 flasks at a density of 3 × 10^5^ cells/flask and allowed to recover for 24 hours in complete media. Following recovery cells were treated with the indicated concentrations of OxC-beta or corresponding concentration of DMSO (vehicle control) for 24 hours. For experiments with THP-1 cells, treatment with 25 ng/mL phorbol myristate acetate (PMA) was used as a positive control. Following treatment cells were harvested via trypsin treatment (for adherent fibroblast and HUVEC cultures) and centrifugation at 300×g for 5 minutes. Cells were then washed by resuspension in 1 mL of phosphate buffered saline (PBS) and centrifugation at 300×g for 5 minutes. Washed cells were resuspended in 200 µL of staining buffer (Dulbecco’s phosphate-buffered saline, pH 7.4, 0.2% bovine serum albumin, 0.09% sodium azide) and the cell density of each suspension was determined by manual counting with a hemocytometer. Suspensions from each treatment group were then transferred, in 100 µL aliquots, into triplicate wells on a 96-well, round bottom microplate (1×10^5^ cells/well) and incubated for 1 hour at 4°C in the presence of 10 µL normal mouse serum (eBioscience 24-5544-94). Cells were then spun at 300×g for 5 minutes, washed by resuspension in PBS and centrifugation, and resuspended in 100 µL of staining buffer. Cells were stained for CD14, TLR-4, or TLR-2 by incubation for 1 hour at 4°C with the following phycoerythrin (PE) conjugated antibodies: anti-human CD14 (eBioscience 12–0149), anti-human TLR-4 (eBioscience 12–9917), and anti-human TLR-2 (eBioscience 12–9922). Cells stained with PE conjugated anti-mouse IgG1 (eBioscience 12–4714) for CD14 or anti-mouse IgG2a (eBioscience 12–4724) for TLR-4 and TLR-2 served as isotype controls. After labeling cells were pelleted by centrifugation at 300×g for 5 minutes and washed once in 200 µL of PBS. Washed cells were then resuspended in 100 µL of stain buffer and fluorescence was determined by flow cytometry using a FACS Array Bioanalyzer (BD Biosciences). FACS data were analyzed using FLOWJO software version 8.8.6. Effects on receptor content were expressed as fold-change in the staining intensity of treated versus control cells. Treatment effect on receptor levels was assessed relative to untreated control cells using a one-way analysis of variance with Tukey’s post-test for multiple comparisons.

### Determination of OxC-beta Effect on Cytokine Profiles in LPS Challenged Monocytes

We hypothesized that OxC-beta may tolerize or prime immune cells to respond to evidence of an invading pathogen based on observations from an earlier animal trial (unpublished). We chose LPS challenge of THP-1 cells as an *in vitro* model to evaluate the potential priming effects of OxC-beta. THP-1 cells were pretreated with the indicated concentrations of OxC-beta (0.1, 0.5, 1.0 µM) for 24 hours, at which point OxC-beta was removed and cells were cultured in the absence of OxC-beta for an additional 5 days. Cells were then challenged with LPS (15 ng/mL) for 24 hours prior to collection of conditioned media for evaluation of cytokine levels. OxC-beta treatment concentrations and the timeline were selected to best model availability of the compound within the host in the earlier animal study.

Analysis of cytokine levels in conditioned media was performed using Endogen Human ELISA kits (Pierce) according to manufacturer’s instructions. Conditioned media were prepared by centrifugation to remove cellular debris and used neat, diluted with complete medium, or concentrated using Nanosep 3K centrifugal concentrators (Pall) to ensure that cytokine levels fell within the linear ranges of each assay. Where appropriate, samples were stored at −80°C and thawed by gradual equilibration at room temperature prior to use. Briefly, 50 µL samples were added to each well of a microplate to which antibody specific to the cytokine of interest had been adsorbed and incubated at room temperature for 1–3 hours. Plates were washed three times to remove nonspecifically bound material and incubated for an additional 1–3 hours with biotinylated antibody specific to the cytokine of interest. After washing, plates were incubated for 30 minutes with streptavidin-horse radish peroxidase reagent followed by an additional washing cycle. Washed plates were incubated for 30 minutes with 3,3′,5,5′-tetramethylbenzidine (TMB) substrate, the reaction was stopped and absorbance was measured at 450 nm (550 nm reference). Reference curves were generated for each cytokine using the supplied recombinant standard.

### Phagocytosis

THP-1 cells were seeded in 96-well plates (1×10^5^ cells/well) in complete media and allowed to recover for 24 hours prior to treatment. Cells were then treated with the indicated concentrations of OxC-beta, the equivalent concentration (v/v) of DMSO (vehicle control), or 25 ng/mL PMA for 24 hours, at which point the compounds were removed and the cells were cultured for an additional 24 hours in complete media containing 15 ng/mL lipopolysaccharide (LPS). Phagocytosis was evaluated using a Vybrant Phagocytosis assay kit (Invitrogen, V6694) based upon the ingestion of fluorescein-labeled *E. coli* (strain K12) bacterial particles. Briefly, treated cells were incubated at 37°C for 5 hours with a 100 µL suspension of fluorescent bioparticles in Hank’s buffered salt solution. Following incubation, the suspension was removed and replaced with 100 µL of 2% trypan blue solution for 1 minute. The trypan blue solution was removed and the number of ingested particles determined using a fluorescence microplate reader (480 nm excitation, 520 nm emission). Wells containing only medium (no cells) served as negative reaction controls against which each experimental replicates were equalized. The OxC-beta effect on phagocytosis was assessed relative to the corresponding DMSO control using Student’s *t*-test.

### Measurement of RAR Agonist Activity

MCF-7 cells were seeded in T-25 flasks at a density of 5×10^5^ cells/flask in 5 mL of complete media. Following a 24-hour recovery period cells were exposed to one of 8 treatment conditions for 24 hours. Three OxC-beta concentrations were tested; 2.5 µM (1.34 µg/mL), 5.0 µM (2.67 µg/mL), and 25 µM (13.4 µg/mL) along with the corresponding three DMSO vehicle controls (0.025%, 0.05%, and 0.25% v/v). Cells treated with 1.0 µM of all-trans retinoic acid served as the positive control while untreated cells served as the negative control.

After 24 hours of treatment cells were trypsinized, collected by centrifugation, and processed for total RNA isolation using the standard protocol of the RNeasy kit (Qiagen 74104). The yield and quality of RNA were verified by absorbance at 260 nm and 280 nm and gel electrophoresis. First strand cDNA was synthesized, according to the manufacturer’s instructions, using Super Script III (Invitrogen 18080-051) reverse transcriptase with random primers and 1 µg of total RNA as template in a reaction volume of 20 µL. Following completion of the synthesis reaction the cDNA was diluted 1∶1 with 20 µL of sterile water and 5 µL of the diluted cDNA was used as template in quantitative real-time PCR (QRT-PCR) assays.

OxC-beta‘s effect on the expression of CYP26A, a known RAR responsive gene [23] was assessed by QRT-PCR using β-actin as a reference gene. Primer sequences for each gene are as follows:

CYP26A Forward TTCAACCGAACTCCTCTTTGGA.

CYP26A Reverse CTTACTCTTCAGCTCTTCTCGCACTT.

β-actin Forward TCATGAAGTGTGACGTGGACATC.

β-actin Reverse CAGGAGGAGCAATGATCTTGATCT.

Reactions were carried out in a total volume of 25 µL containing 12.5 µL of 2 x Platinum^®^ SYBR^®^ Green qPCR Super Mix-UDG (Invitrogen 11733-046), 0.5 µL each of forward and reverse primer (10 µM), 2.5 µL of diluted cDNA, and 9 µL sterile water. All reactions were run in triplicate on a Rotor-Gene 6500 HRM instrument (Corbett Life Sciences) running software version 1.7 with the following cycling parameters: 10 minutes at 50°C, 5 minutes at 92°C, 40 cycles of (45 seconds at 95°C, 25 seconds at 62°C, and 30 seconds at 70°C). The presence of a single amplified product in each reaction was verified by melt analysis.

Expression of CYP26A was calculated relative to β-actin using the two standard curve method. The mean ratio of relative expression was calculated based on 3 replicate experiments and treatment effects were tested for statistical significance by one-way ANOVA with Tukey’s test for multiple comparisons.

### CD14 Receptor Assay

The CD14 receptor assay is based on research demonstrating that OxC-beta treatment of THP-1 cells induced an increase in the surface content of CD14 receptors. The procedure for the receptor assay is described below.

Human THP-1 monocytes were seeded (5×10^5^ cells/well) in 3 mL of media into 6-well culture plates and allowed to recover for 24 hours prior to treatment. Following recovery cells were treated for 24 hours with 0, 1.0, 2.5, 5.0, and 10 µM of the test compound before being processed for CD14 membrane content via FACS analysis. Cells were harvested for FACS analysis by centrifugation at 300×g for 5 minutes and then washed once by resuspension in 1 mL of 1 x phosphate buffered saline (PBS) and centrifugation at 300×g for 5 minutes. Washed cells were next resuspended in staining buffer (1 x PBS, 0.1% BSA) and the cell density of each suspension was determined by manual counting with a hemocytometer. Suspensions from each treatment group were then transferred, in 100 µL aliquots, into triplicate wells on a 96-well, round bottom microplate (1×10^5^ cells/well). 10 µL of normal mouse serum was then added to each well and cells were incubated for 30 minutes at 4°C. Cells were then stained for CD14 by the addition of 10 µL (0.2 µg) of phycoerythrin (PE) conjugated human anti CD14 antibody (eBioscience 12–0149) and incubation for 1 hour at 4°C. Cells stained with PE conjugated anti-mouse IgG1 (eBioscience 12–4714) served as isotype controls. Following incubation with the antibodies cells were pelleted by centrifugation at 300×g for 5 minutes and washed once in 200 µL of wash buffer. Following washing cells were resuspended in 100 µL of stain buffer and evaluated for CD14 content by FACS analysis using a FACS Array Bioanalyzer (BD Biosciences). FACS data were analyzed using FLOWJO software version 8.8.6. Test compound effects on CD14 levels were expressed as fold-change in the number of CD14 positive cells relative to untreated controls.

### Effect of OxC-beta Feeding on Gut Immune Receptor Expression

#### Animals

Twenty-eight Balb/c mice, 2-weeks of age with body weights ranging from 18–20 g, were purchased from Charles River Laboratories, Canada. Mice were placed in appropriate holding facilities at the Atlantic Veterinary College (Charlottetown, PE, Canada) and were handled according to the institutional guidelines for animal experimentation. The animal use protocol for this study was approved by the University of Prince Edward Island’s Animal Care Committee. Mice were housed under constant environmental conditions with 12 hours light/12 hours dark cycles and ad libitum access to food and water. An acclimation period of 1-week was completed prior to beginning the study. On day 0 mice were distributed into 7 cages with 4 mice per cage. Each cage contained 3 treated mice and 1 control mouse. Individual mice within each cage were identified by banding the tail using a felt marker. Treated mice were divided into low dose (1 mg OxC-beta/kg body weight) or high dose (10 mg OxC-beta/kg body weight) groups and each dose group was then further divided into either a 2-week or 4-week treatment period. OxC-beta or vehicle control was administered daily by oral gavage for the duration of the study. Gavage solutions were prepared by diluting stock solutions of OxC-beta in ethanol with saline to give the appropriate dose of OxC-beta in 1% ethanol. In order to account for growth of the mice during the trial mice were weighed every second day and the volume of gavage solution administered was adjusted to maintain a constant dose.

### Processing of Tissue Samples

At the conclusion of the 2 and 4-week supplementation periods mice were euthanized and necropsied for isolation of the small intestine. Following removal of the digestive contents the intestinal tissue was opened with a lateral incision and the mucosal surface was gently rinsed with saline. The tissue sample was next separated into 3 sections, ileum, jejunum and duodenum and each section was fixed with 10% buffered formalin for 16 hours. Following fixing, tissue samples were embedded in paraffin blocks and were sectioned for preparation of slides.

### Immunohistochemical Staining for CD14 and TLR-4

Tissue sections were stained using primary polyclonal antibodies against murine CD14 (Abcam ab25090) or TLR-4 (Abcam ab53629) and the VectaStain Elite ABC kit (Vector Laboratories PK 6100 and PK 6101) according to the manufacturer’s instructions. Bound antibodies were detected by incubation in DAB peroxidase substrate solution (Vector Laboratories SK-4100). Cover slips were applied to slides using permount medium and slides were allowed to dry before analysis using light microscopy. Slides were analyzed by observation under a light microscope at 10x, 20x and 40x magnifications.
